# Beneficial Effect of Antibodies against β- Secretase Cleavage Site of App on Alzheimer’s-Like Pathology in Triple-Transgenic Mice

**DOI:** 10.1371/journal.pone.0046650

**Published:** 2012-10-10

**Authors:** Inna Rabinovich-Nikitin, Idan S. Rakover, Maria Becker, Beka Solomon

**Affiliations:** Department of Molecular Microbiology and Biotechnology, George S. Wise Faculty of Life Sciences, Tel-Aviv University, Tel-Aviv, Israel; Massachusetts General Hospital and Harvard Medical School, United States of America

## Abstract

The toxicity of amyloid β and tau, the two hallmark proteins in Alzheimer’s disease (AD), has been extensively studied individually. Recently new data suggest their possible interactions and synergistic effects in the disease. In this study, we investigate the ability of antibodies against the β secretase cleavage site on APP, named BBS1, to affect tau pathology, besides their well established effect on intracellular Aβ and amyloid load. For this purpose we treated the triple transgenic mice model of AD (3x Tg-AD) with mAb BBS1 intracerebroventricularly, using mini osmotic pumps for one month. The experimental data demonstrated reduction in total and phosphorylated tau levels, explained by significant reduction in GSK3β which phosphorylates tau on sites recognized by antibodies against PHF1 and AT-8. The treatment increased the cognitive capabilities and reduced the brain inflammation levels which accompany AD pathology. The data showing that tau pathology was significantly reduced by BBS1 antibodies suggest a close interaction between tau and Aβ in the development of AD, and may serve as an efficient novel immunotherapy against both hallmarks of this disease.

## Introduction

Alzheimer’s disease (AD) is a progressive mental disorder causing impairment of memory and other cognitive functions [1], [2]. There are two main pathological hallmarks of AD: amyloid plaques and neurofibrillary tangles.

Neurofibrillary tangles formed from the microtubule associated protein, tau, are localized in neuronal axons and have the ability to promote microtubule assembly by stabilizing its structure [3], [4]. The phosphorylation of tau plays a physiological role in regulating the affinity of tau for microtubules, being a substrate for many kinases [5], such as glycogen synthase kinase 3β (GSK3β), well known as tau kinase I, a serine/threonine kinase, that is widely expressed in the developing and adult brain and is most abundant in neurons. The phosphorylation of tau by GSK3β, together with other kinases, inhibits the ability of tau to assembly the microtubule and causes the polymerization of tau into the toxic neurofibrillary tangles[6]–[8].

The amyloid plaques in the brain in AD contain the Aβ peptide. The amyloid beta deposits are produced from a proteolytic processing of the amyloid precursor protein (APP). In the amyloidogenic pathway APP is first cleaved by the β-secretase cleaving enzyme (BACE1), generating the soluble APPβ fragment and a membrane-bound APP carboxy-fragment- CTFβ. The CTFβ fragment, which consists of 99 amino acids, is subsequently cleaved by the γ-secretase cleaving enzyme generating a residue of 40 or 42 amino acids [9], [10].

BACE1 is a 501 amino acid transmembrane aspartyl protease expressed in all tissues and highly expressed in the brain [11], [12]. This protease has a substantial role in initiating the amyloidogenic pathway, thus promoting it as a prime target for drug discovery in AD. There are some rising concerns regarding the inhibition of BACE1 including the fact that BACE1 also processes other substrates, thus might cause toxicity by affecting other natural immunological and neurological targets in physiological processes besides the inhibition of APP processing itself [13], [14].

In order to overcome the challenges raised from inhibiting BACE1 we developed a different approach using site-directed antibodies to inhibit the initiation of APP processing. These antibodies block the BACE cleavage site on the APP substrate, thus interfering with APP-BACE interaction. The monoclonal antibodies (mAb), called blocking β site 1 (BBS1), were raised against amino acids on APP that contain the BACE cleaving site. The mAb BBS1 was generated against a multiple antigenic peptide (MAP) displaying 8 copies of the half Swedish mutation in which the M670L mutation was introduced (MAP-[ISEVKLDA]_8_). The mechanism of action of mAb BBS1 is based on binding of the antibody at the cell surface before internalization to the early endosome where BACE cleaves the APP. This mode of action was previously demonstrated by using a cellular model overexpressing the wild-type human APP751 isoform. The BBS1 antibodies incubated with the cells were co- internalized into the early endosomes after only 2 min of incubation as well as to the lysosomal compartment after 30 min of incubation [15].

Previous experiments with the mAb BBS1 demonstrated reduction in Aβ levels in both cellular and animal models. In Chinese hamster ovary cells over-expressing the wild type APP751 isoform, mAb BBS1 was shown to decrease both secreted and intracellular Aβ levels, as well as CTFβ levels [15]. The in vivo capabilities of mAb BBS1 were demonstrated in both Tg2576 and London mutation mice models. Long term systemic administration with mAb BBS1 to the Tg2576 mouse model of AD improved cognitive function, and reduced brain inflammation and microhemorrhage without inducing peripheral autoimmunity [16]. Systemic treatment with the same antibody in the London mutation mouse model resulted in reduced levels of amyloid burden, insoluble Aβ40 and Aβ42 and membrane-associated Aβ oligomers [17]. The ability of BBS1 treatment to reduce inflammation, as shown in these previous studies, proves that the treatment is safely to use and does not include any side effects.

Here, we investigate the feasibility of intracerebroventricular (ICV) administration of the mAb BBS1 in the triple transgenic mice model (3xTg-AD) over-expressing human APP_Swe_, tau_P301L_ and PS1. We found that, after one month of continuous mAb BBS1 administration with mini-osmotic pumps, cognitive functions were improved. The treatment succeeded in affecting tau pathology via GSK3β; total and phosphorylated tau and brain inflammation were dramatically decreased in addition to reduction in total Aβ burden. The present study might shed a light on the interaction between Aβ and tau and emphasize the immunotherapeutic significance of mAb BBS1 in AD treatment.

## Results

In this study we examined the ability of mAb BBS1 to affect tau and Aβ levels and thus to evaluate the effect of blocking the BACE cleavage site on APP for AD pathology. The 3xTg-AD mice used in this study develop both Aβ plaques and tau aggregates in an age-dependent and brain-region-specific manner; representing a close model of disease progression in humans. Mice were implanted at 17 months of age with mini-osmotic pumps, pumping 7.5 mg/kg of mAb BBS1 or control Ab OK1 at a rate of 0.25 µl per hour for 28 days. Towards the end of the treatment period, mice were evaluated in a cognitive test and sacrificed immediately afterwards. The brains were divided for biochemical and histological analysis to follow the effect of the treatment on the neuropathological hallmarks.

### mAb BBS1 Treatment Improves Cognitive Function

**Figure 1:**
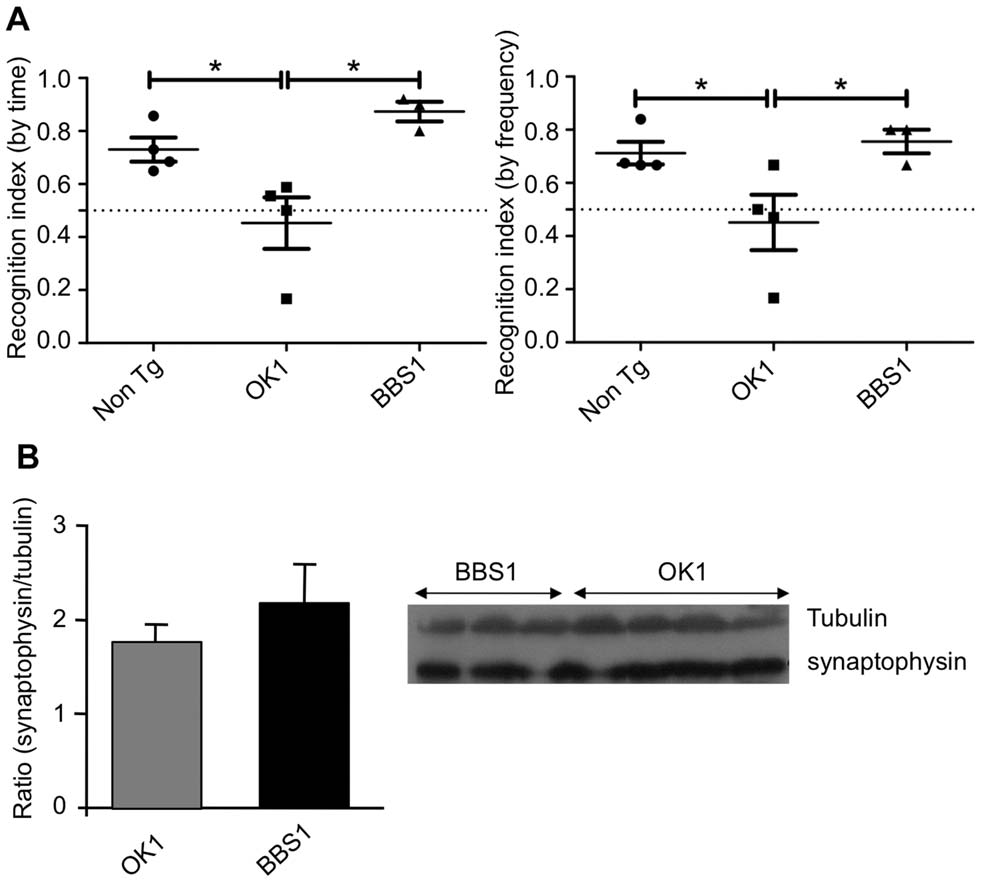
Evaluation of BBS1 effect on mice cognition. Cognitive improvement was assessed using the object recognition test and synaptophysin levels. (**A**) Mice were subjected to a three-day object recognition test. The time and frequency that each mouse spent near the novel object on the third day were recorded. The recognition index was defined as [time or frequency spent near the novel object/total time or frequency spent near both objects]. (**B**) After sacrifice, mice homogenized brains were examined for synaptophysin levels using immunoblot analysis.

Cognitive function following mAb BBS1 treatment was demonstrated using the object recognition test. Mice were trained for 2 days, first in an empty arena and then with one object. On the third day mice were exposed to a novel object in addition to the familiar one, and the frequency and time spent near the novel object was recorded. The group treated with control mAb had no preference for either of the objects, thus its recognition index was ∼0.5 for both frequency and time spent near the novel object. The group treated with mAb BBS1 performed significantly better than the OK1 group (RI at time = 0.88, RI at frequency = 0.8) and reached the level of the non-transgenic control mice (RI at time = 0.75, RI at frequency = 0.8) (Fig. 1A).

Regional loss of synapses related to cognitive impairments is a characteristic of Alzheimer’s disease [18]. The treatment was beneficial as shown by a 24% increase in synaptophysin level in mice treated with mAb BBS1 (Fig. 1B).

### BBS1 Antibody Reduced Total Aβ Accumulation and Average Plaque Size with no Change in the Number of Plaques

**Figure 2:**
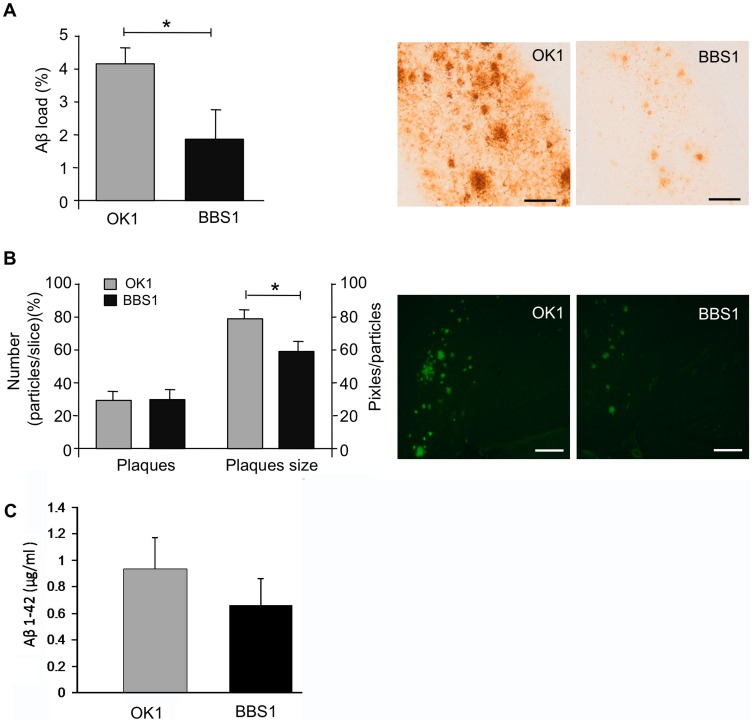
Evaluation of BBS1 treatment on Aβ content. BBS1 treatment reduced levels of total Aβ and average plaque size but did not affect the number of plaques. (**A**) Immunostaining with 4G8 antibody for quantifying total Aβ levels in 3xTg-AD mice. Scale bar is 100 µm. (**B**) Modified Thio-S staining of dense core plaques in the subiculum. Scale bar is 20 µm. (**C**) Sandwich ELISA technique for assessing Aβ 1–42.

Aβ is derived from the transmembrane APP by consecutive proteolytic cleavages [9]. Accumulation of Aβ peptides leads to deposition of senile plaques in the extracellular space, eventually causing neuronal death [19]. Treatment with mAb BBS1 relative to treatment with control mAb demonstrated a significant reduction of 56% in total Aβ burden detected with immunostaining with the 4G8 antibody (Fig. 2A).

The modified Thio-S staining method allows observing dense core senile plaques in the subiculum [20]. Using this method we observed no reduction in the number of plaques among the treated mice, but a clear reduction of 24% in the average dense core plaque size following mAb BBS1 relative to control mAb (Fig. 2B).

Using the sandwich ELISA technique, we detected Aβ 1–42 levels in brain homogenates. Brain extracts were captured between the 266 antibody, recognizing the central region of Aβ at residues 17–28, and 21F12 antibody which specifically precipitates Aβ peptides ending at position 42. Measuring the absorption of the experimented plate at 405nm revealed 24% decrease in Aβ 1–42 levels following mAb BBS1 relative to control mAb (Fig. 2C).

### BBS1 Treatment Affects Tau Pathology and GSK3β Levels

**Figure 3:**
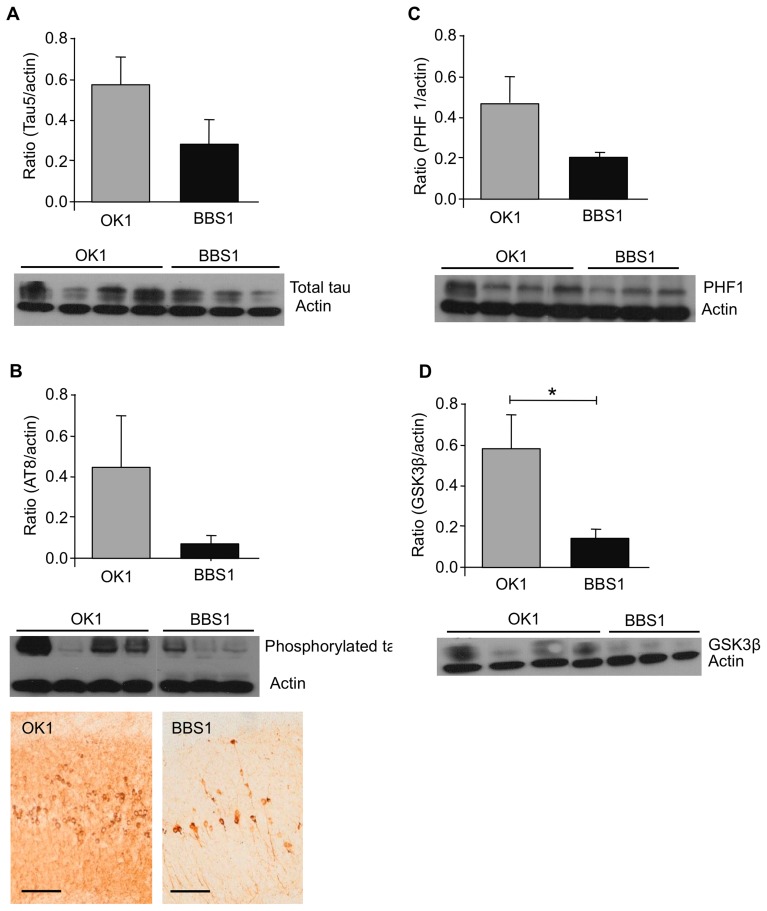
BBS1 reduced levels of total tau, phosphorylated tau and GSK3β. Reduced levels of total tau and phophorylated tau in mice treated with mAb BBS1 may be mediated by reduced levels of GSK3β. (**A**) Total tau levels were quantified using tau 5 antibody in immunoblot. (**B**) Levels of phosphorylated tau at S199/S202/T205 positions were quantified using AT8 antibody. Scale bar is 500 µm. (**C**) Levels of paired helical filaments of tau phosphorylated at S396/S404 positions were quantified using PHF1 antibody in immunoblot analysis. (**D**) GSK3β levels were quantified by immunoblot analysis.

Tau plays an important role in stabilizing the microtubules. Aberrant phosphorylation of tau results in an impairment in the normal function of tau and formation of neurofibrillary tangles [21]. Mice treated with mAb BBS1 relative to control mAb showed a decrease of 51% in total tau levels (Fig. 3A) and 80% of phosphorylated tau levels (Fig. 3B), recognized in both biochemical and histological analysis with AT-8 antibody. Antibody against PHF1 measures paired helical filaments of tangles phosphorylated at S396/S404 [22]. Immunoblot analysis with this antibody revealed a 56% decrease in tau phosphorylated at those positions among mice treated with mAb BBS1 relative to control mAb (Fig. 3C).

GSK3β, a serine/threonine protein kinase, has a key role in phosphorylating tau and disassociation from the microtubule [23]. Mouse models displaying increased expression of GSK3β result in high levels of phosphorylated tau [24]. The total GSK3β levels were significantly decreased by 74% (Fig. 3D).

### Evaluation of Brain Inflammation in Mice Treated with BBS1

**Figure 4:**
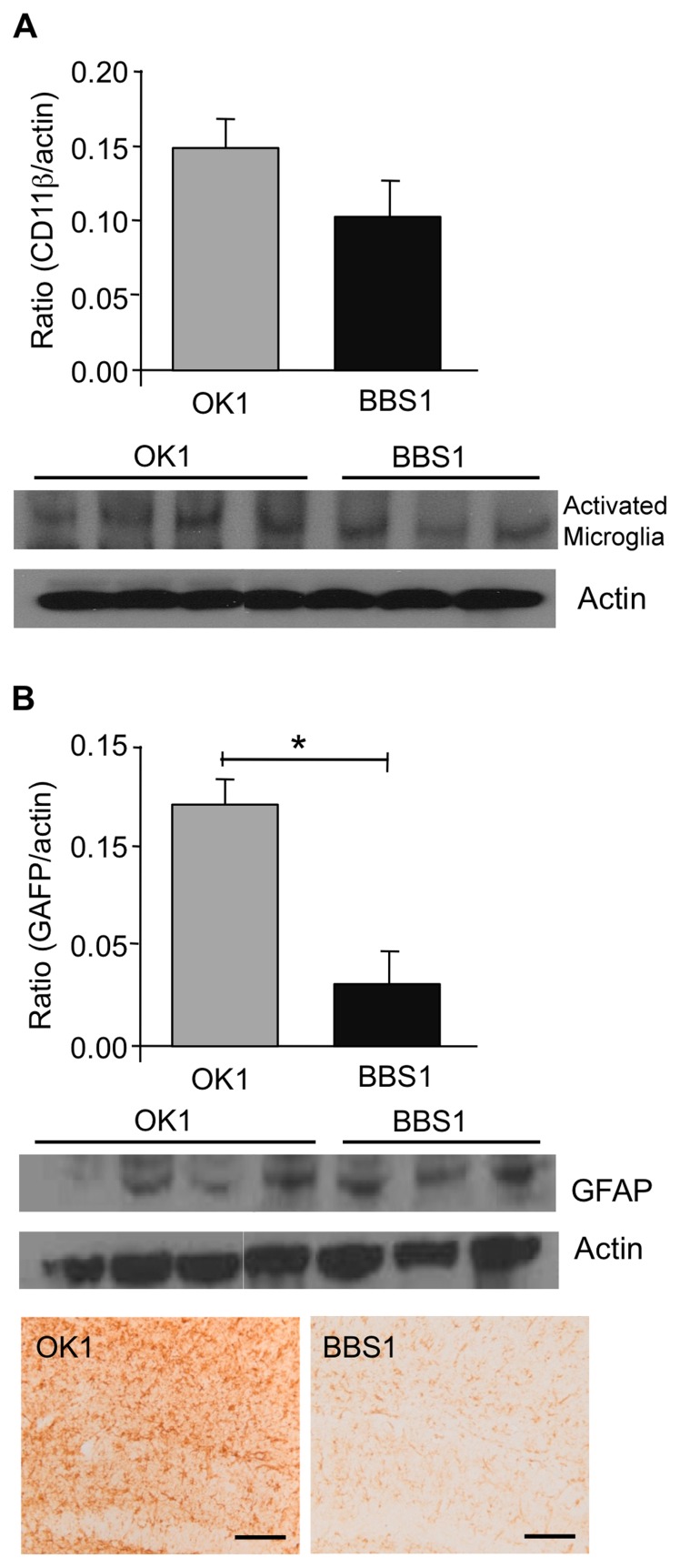
Evaluation of BBS1 effect on inflammation. BBS1 treatment reduced levels of activated microglia and reactive astrocytes. (**A**) Activated microglia levels were evaluated with the CD11b antibody in immunoblot analysis. (**B**) GFAP levels were measured as indicative of the astrocytes reactive levels in both immunostaining and immunoblot analysis. Scale bar is 100 µm.

Damaged neurons, deposited Aβ and neurofibrillary tangles provide stimuli for inflammation in AD brain [25]. Glial cells, especially microglia, become reactive in the presence of deposits of Aβ. It was found that in the 3xTg-AD mice model there is increase in the reactive microglia in accordance with increase in the amyloid burden. CD11b is highly expressed on reactive microglia, making it a suitable marker for levels of active microglia [26]. Biochemical analysis with anti-CD11b antibody demonstrated a 31% decrease in this marker among the treated mice (Fig. 4A) and hence a reduction in the activated microglia.

Severity of AD pathology correlates strongly with the density of astrocytes. The glial fibrillary acidic protein (GFAP) is upregulated in activated astrocytes [27]. Levels of GFAP were tested in both immunohistochemical and biochemical methods and revealed a significant decrease of 74% in the activated astrocytes levels in mice treated with mAb BBS1 relative to control mAb (Fig. 4B).

## Discussion

In order to overcome some of the drawbacks that may arise from direct inhibition of BACE1, we developed a novel approach to inhibit Aβ production via antibodies against the BACE cleavage site of APP. These antibodies bind both wild-type and Swedish-mutated APP expressed in transgenic mouse brain tissues and do not bind any form of Aβ peptides [28]. Administration of these antibodies to the cells [15] and to transgenic mice models of AD [16], [17] resulted in a considerable decrease in intracellular Aβ levels including toxic oligomers. The relevance of intraneuronal accumulation of mainly Aβ42 as an early event in AD pathogenesis suggests that this approach may be applicable as a novel therapeutic strategy in AD treatment.

The AD transgenic mouse model used in this work is the 3xTg-AD mice model harboring the human APP_swe_ and human tau_P301L_ genes in PS1 knockin mouse; these mice develop Aβ plaques and tau inclusions as well as a remarkable level of accumulation of intracellular Aβ. The initial characterization of this model demonstrated a close relationship between intraneuronal Aβ immunoreactivity, plaques formation, and tau pathologies [29]. Since this mouse model mimics closely the pathological state in AD patients, it enables an investigation of immunization on both plaques and tangles formation and their possible interaction.

The one-month ICV administration of mAb BBS1 via mini-osmotic pumps to 3xTg-AD mice was examined in the object recognition test which measured non-spatial long-term memory in mice. The test is based on the spontaneous tendency of rodents to explore a novel object longer than a familiar one. The assay resulted in significant improvement of mice cognitive abilities and close to the cognitive level of control non-transgenic mice. There is a 24% increase in the associated pre-synaptic vesicle protein, synaptophysin, which indicates synaptic plasticity, regulates short and long term memory and correlates with cognitive decline in AD [30]. This result correlates with the observed cognitive improvement, suggesting that mAb BBS1 treatment inhibits the synaptic destruction evolved with the disease.

Recent studies are trying to decode the possible synergistic effects between Aβ and tau, suggesting three possible modes of interaction. The first mode claims that Aβ drives tau pathology by causing its hyperphosphorylation, which in turn mediates toxicity in the neurons. In the second mode, tau mediates the toxicity of Aβ which is critically dependent on the presence of tau. The third mode asserts that both Aβ and tau target various cellular processes or organelles and in this manner enhance the mutual toxic effects [31]. In the 3xTg mice model, Aβ and tau impair mitochondrial respiration related to different complexes; tau impairs complex I and Aβ blocks complex IV of the respiratory chain, leading to increase mitochondrial impairment, compared with mice overexpressing tau or Aβ alone [32]. Examination of total tau levels demonstrated a 51% decrease in steady-state tau expression. Tau phosphorylation plays a critical role in stabilizing microtubules and promoting their assembly and dynamic stability. Hyperphosphorylation of tau mediates its mislocalization and subsequent synaptic impairment. Previous study indicates that the locus of early synaptic malfunction caused by tau resides in dendritic spines [33]. We report reduction of >50% in tau phosphorylated at positions S199/S202/T205 and in brain-phosphorylated tau at positions S396/S404.

We found a significant decrease in total GSK3β levels, a major tau kinase after mAb BBS1 treatment. In pathological phosphorylation of tau, GSK3β phosphorylates tau at different positions; thus the major reduction in phosphorylated tau levels demonstrated with those antibodies can be attributed to the reduction in GSK3β levels. GSK3β, a proline-directed serine/threonine kinase, is expressed at high levels in the CNS. Its role is to phosphorylate cytosolic proteins, including tau protein. In the 3xTg-AD mice model, it has been previously observed that activation of GSK3β is correlated with increase in tau phosphorylation [34]. Increased levels of GSK3β have been found in AD brains, and active GSK3β was found to be accumulated in pre-tangle neurons [35].

The administration of mAb BBS1 in the 3xTg-AD mice model was associated with a >50% reduction in total Aβ levels, both intraneuronal and extracellular. This activity of mAb BBS1 was already shown in the Tg2576 and London mutation transgenic mice [16], [17] and it strengthens the evidence that mAb BBS1 is capable of inhibiting the production of the different fragments of Aβ. Moreover, intraneuronal Aβ, one of the APP cleavage products, has been suggested to play a role in cellular damage in early stages of AD, before plaques begin to accumulate. Intraneuronal Aβ was found to affect various cellular systems by pathological damage, including to mitochondria, proteosome and synapses [36]–[40]. The inhibition of Aβ production by immunotherapy with mAb BBS1 was found to attenuate both intraneuronal and extracellular Aβ toxicity. The results showed no difference in the number of plaque particles but a significant decrease in average plaque size, indicating that mAb BBS1 has no influence on disaggregating existing plaques but it inhibits further assembly of the existing plaque, due to the deprivation of APP processing. Levels of the toxic form of amyloid deposits, Aβ 1–42, were assessed using sandwich ELISA. The experiment revealed a decrease in Aβ 1–42 levels in BBS1 treated mouse brains, again demonstrating the ability of mAb BBS1 to inhibit the different cleavage products of APP, especially those considered more toxic in AD.

Genetic studied of FAD have been considered the strongest evidence supporting the amyloid hypothesis; however, there is also evidences that FAD mutations in APP and presenilins might act through amyloid-independent mechanisms as well [41].

Indeed, Aβ may not be the only active component of AD neurotoxicity. It may involve other proteolytic APP fragments such as APP-βCTF and/or AICD. APP-βCTF can form channels in membranes and induce inward currents of ions like sodium and calcium. βCTF turns neurons to be more vulnerable to glutamate-induced excitotoxicity, and part of βCTF forms a partial β sheet that also causes self-aggregation and toxicity [42]. A recent intriguing study suggested a possible mechanism of interaction among GSK3β, beta amyloid and tau [43]. The hypothesis suggested in this study claims that AICD fragments, the 6kDa C terminus fragments of APP, cause transcriptional activation after trans-localization into the nucleus, suggesting a role of AICD in gene regulation. AICD fragments, generated following γ-secretase cleavage of APP, and the C31 fragments generated from further cleavage by caspase 8 and 9, trans-locate into the nucleus accompanied by other transcription factors and induce the transcription of GSK3β. The in vivo evidence for this hypothesis is clear nuclear staining of APP-CT, but not N-terminal fragment of APP, which has been detected, colocalized with Fe65 in post mortem AD brain, in addition to increased levels of GSK3β and active form of GSK3β which have been found to be accumulated in pretangled neurons. According to this theory, inhibition of APP cleavage by mAb BBS1 administration results in decreased levels of the different Aβ cleavage products, including AICD and C31, and thus interfere with the co-localization of these products to the nucleus. GSK3β transcription is delayed and the phosphorylation of tau is reduced.

As been described earlier [43] and in further studies[44]–[46], GSK3β, as well as other kinases, affect APP processing and the derived neurotoxic Aβ deposits. Therefore, a therapy that is capable of reducing tau phosphorylation and neurofibrillary pathology might have neuro-protective potential in AD by reducing amyloidogenesis.

Inflammation occurs in pathologically vulnerable regions of the AD brain. In the presence of aggregated Aβ, microglia becomes active, forming clusters at sites of deposited amyloids and neuritic plaques and so contributing to amyloid clearance. Like microglia, astrocytes also become active proximately to aggregated plaques and together they compose the inflammatory response [47].The activated microglia levels in response to mAb BBS1 treatment examined in this study was decreased by 31%. Reactive astrocytes expression showed a 74% decrease in both immunohistochemical and biochemical analysis. A recent study showed that the number of GFAP-positive astrocytes is closely correlated with memory impairment and neuronal loss, meaning that the inflammatory response of astroglial cells is a key event in memory disruption and neuronal cell death. Based on these findings, it can be argued that immunotherapy with mAb BBS1 is capable of decreasing the inflammation involved in the pathology of the disease and accordingly improve memory. This decrease in inflammation following immunotherapy with mAb BBS1 compared to control mAb provides for an increased margin of safety relative to other immunotherapies for which brain inflammation has been observed in clinical trials [48].

In conclusion, the administration of mAb BBS1 to 3xTg-AD mice not only improves the cognitive function of the mice, but also lowers the levels of total Aβ, total tau and phosphorylated tau, and moderates the inflammatory response. Moreover, the results shown in this study suggest a possible mechanism of the pathological interaction between Aβ and tau in which delaying Aβ production results in a significant decrease in phosphorylated and non phosphorylated tau levels. The multi-faceted action of mAb BBS1 provides a novel and pleiotropic mechanism for AD immunotherapy with an increased margin of safety over other immunotherapies and enzyme-inhibitory small molecules.

## Materials and Methods

### Ethics Statement

The animal study was approved and performed according to the protocols of the Tel-Aviv University Animal Care and Use Committee (permit number L12-011). All surgery was performed under ketamine xylazine anesthesia, and all efforts were made to minimize suffering.

### Transgenic Mice

17-month-old, female 3xTg mice (kindly received from Prof. Frank M. LaFerla) expressing concomitantly the PS1_M146V_, APP_Swe,_ and tau_P301L_ transgenes as described previously [29] were used for this study. Briefly, single cell embryos harvested from homozygous mutant PS1M146V knockin (PS1-KI) mice were microinjected with human APP_Swe_ and human tau_P301L_ genes, both under control of the mouse Thy1.2 regulatory element.

### Treatment and Implantation of Alzet Mini Osmotic Pumps

Mice were implanted with Alzet mini-osmotic pumps, model 2004 (Palo Alto, CA), as previously described [49], pumping its content in a rate of 0.25 µl per hour for 28 days. Pumps were loaded with either 7.5 mg/kg/month BBS1 antibody (n = 3) or with the same dose of a non relevant OK1 isotype-matched antibody, designed against bacteriophage (n = 4), in artificial cerebral-spinal fluid (CSF). The mini osmotic pumps were adjusted ICV of the right hemisphere.

### Behavioral Analysis

After one month of treatment, non-spatial memory was tested using the object recognition test, according to the protocol previously described[50]–[52]. Briefly, on day 1 each mouse was placed in an empty arena (50×50×20 cm) for 5 minutes of exploration. On day 2, mice were placed in the same arena containing one object, for 5 minutes of training. On day 3, mice explored for 5 minutes the same arena, containing the familiar object from day 2 and a new object. The time and frequency each mouse spent near the novel object was recorded. The ratio between the time and frequency each mouse spent around the novel object to the total time and frequency spent at both objects was defined as the recognition index.

### Protein Extraction and Immunoblots

After the 28 days treatment mice were anaesthetized IP with 100 mg/kg Ketamine (Fort Dodge, USA) and 20 mg/kg Xylazine (Merck, Germany), and then intracardially perfused with saline. Brain tissues were cut in half sagittally, and the left hemisphere, was frozen in liquid nitrogen and stored at −70°C until homogenization. Brain extracts were prepared by homogenizing the hemisphere in T-per (Pierce, USA) extraction buffer complemented with protease inhibitor tablets (Complete Mini Protease Inhibitor Tablets, Roche), phosphatase inhibitor cocktail tablets (phosSTOP, Roche), 0.5% Triton-100, 1% sodium deoxy-cholate and 3% SDS. Following homogenization, brain extracts were sonicated and centrifuged for 10 minutes at 100,000 g and the supernatant was collected for protein concentration determination using BCA protein assay kit (Thermo, USA). Equal amounts of extracted protein (80 µg) were loaded on a 15% polyacrylamide gel and transferred to nitrocellulose membrane. Membranes were blocked overnight with 5% skim milk in TBS-T (0.3% Tween 20), following 1 hour of incubation with primary antibodies (Table 1) and one hour incubation with secondary antibodies. Immunoblots were developed with the EZ- ECL detection kit (Biological Industries, Israel), and quantitative densitometric analysis was performed using the densitometric software EZQuant-Gel 2.12.

**Table 1:** Antibodies used in Western Blot.

Antibody	Immunogen	Host	Dilution	Source
Synaptophysin	Synaptic vesicles	Rabbit	0∶73611	Dako
TAU -5	Central region of microtubuleassociated protein	Mouse	0∶73611	Calbiochem
AT-8	Phospho S199/S202/T205	Mouse	0∶73611	Innogenetics
PHF1	Phospho S396/S404	Mouse	0∶73611	Dr. P Davies
GSK- 3β	Glycogen Synthase Kinase 3β	Mouse	0∶38889	Sigma- Aldrich
GFAP	Glial Fibrillary Acidic Protein	Rabbit	1∶10000	Dako
CD11b	Mac- 1	Rabbit	0∶38889	Novus Biologicals
Actin	N- terminal β cytoplasmic actin	Mouse	3∶51389	Sigma- Aldrich
Tubulin	C- terminal β tubulin	Mouse	1∶25000	Sigma- Aldrich

Dako, Denmark; Calbiochem, San Diego; Innogenetics, Belgium; Sigma Aldrich, USA; Novus Biologicals, USA.

### Sandwich ELISA for Detection of Aβ 1–42

Microtiter plates (Nalge Nunc) were coated with antibody 266 (ELAN, Ireland), recognizing Aβ 17–28, diluted 1∶500 in 0.1M Na2CO3 (pH 9.6) overnight at 4°C. On the next day, plates were washed twice with PBS-T (0.05% Tween 20), followed by two washes with PBS, and blocked with 3% BSA in PBS, for 3 h at 37°C. Brain homogenates were added to the plate, and incubated overnight at 4°C. Biotinylated 21F12 (ELAN, Ireland) diluted 1∶2500 in 1% BSA was added to each well and incubated for 3 hours at 37°C. Extra-avidin Alkaline phosphatase conjugated (Sigma, USA) diluted 1∶30,000 in 1% BSA was added for 1 hour at 37°C, then 1 tablet of p-nitrophenyl phosphate (pNPP) substrate (Sigma, USA) in developing buffer (1M Diethanolamine 1M, 0.0001% MgCl_2_, 0.0002% sodium azide in DDW, pH 9.8) was added. The dephosphorylation of the substrate was measured at 405 nm after 1, 4 and 16 hours following application to the plate.

### Immunohistochemistry

Right hemisphere of the brain was fixed in 4% formalin in PBS for 48 hours. After fixation brains were immersed overnight in 30% sucrose in PBS. Brains were frozen with dry ice and kept at −70°C. 25 µm free floating coronary sections were obtained using a cryostat (LEICA CM 1900, Germany). Sections were stained with 0.01% Thio-S in 50% ethanol for 8 minutes in the dark, followed by 2 washes in 80% ethanol for 10 seconds each and rinsing in DDW. For purpose of immunohistochemistry, sections were treated with 3% H_2_O_2_ in absolute methanol for 15 minutes, following incubation in 90% formic acid for 4 minutes. Sections were blocked with Ultra V block (Thermo, USA) for 10 minutes and with 10% fetal calf serum in PBS for 30 minutes. For total Aβ staining a biotin blocker (AbD serotec, UK) was also applied for 30 minutes. The primary antibodies were applied for overnight incubation at 4°C as follows: biotinylated Aβ antibody 4G8 (Covance, USA) 1∶10,000; phosphorylated tau AT8 (Innogenetics, Belgium) 1∶250; GFAP (Dako, Denmark) 1∶500. Super picture poly HRP conjugate (Invitrogen, USA) was applied for 15 minutes for 4G8 and AT8 staining and HRP-conjugated goat anti-rabbit IgG Fc antibody 1∶1000 (Jackson ImmunResearch) was applied for 1 hour for GFAP staining. Sections were visualized using DAB chromogen substrate (Invitrogen, USA), dehydrated in graded alcohol, cleared in xylene and coverslipped with enthelan (Merck, Germany).

Images were captured by a CCD color video camera (ProgRes C14, Jenoptic, Jena, Germany) attached either to a Leica MZ6 binocular (Leica, Germany) for GFAP and 4G8 staining or to a Leica DMLB microscope (Leica, Germany) for AT8 and modified Thio-S staining. In both cases, Image-J Software (NIH, freeware) was used for all analyses.

### Statistical Analyses

All data presented as the mean ± SEM, was subjected to Mann Whitney significance test and analysis of variance (ANOVA). The significant P value was set to 0.05 for all statistical analyses.
